# Dietary Determinants of Diabetes Prevalence: A Cross-Sectional Study in the Hungarian Population

**DOI:** 10.3390/nu18050731

**Published:** 2026-02-25

**Authors:** Zsuzsa Emma Hajzer, Flóra Mária Petróczki, Eszter Vargáné Faludi, Csaba Oláh, József Prokisch, Amr Sayed Ghanem

**Affiliations:** 1 Department of Dietetics, Faculty of Health Sciences, University of Debrecen, 4028 Debrecen, Hungary; hajzer.zsuzsa@etk.unideb.hu (Z.E.H.); petroczki.flora@etk.unideb.hu (F.M.P.); 2 Department of Epidemiology, Faculty of Health Sciences, University of Debrecen, 4028 Debrecen, Hungary; faludi.eszter@etk.unideb.hu; 3 Neurosurgery Department, Borsod County University Teaching Hospital, 3526 Miskolc, Hungary; olahcs@gmail.com; 4 Faculty of Agricultural and Food Sciences and Environmental Management, Institute of Animal Science, Biotechnology and Nature Conservation, University of Debrecen, 4032 Debrecen, Hungary; jprokisch@agr.unideb.hu

**Keywords:** diabetes mellitus, dietary intake, nutritional epidemiology, socioeconomic determinants, lifestyle risk factors, Hungary, public health nutrition, cross-sectional analysis

## Abstract

**Background/Objectives**: Diabetes mellitus represents a major public health burden in Hungary and is associated with sociodemographic and lifestyle characteristics. This study examined cross-sectional associations between habitual dietary intake and self-reported diabetes prevalence in a nationally representative Hungarian sample. **Methods**: Data from 5603 participants aged ≥15 years in the 2019 European Health Interview Survey (EHIS) were analyzed. Survey-weighted logistic regression models assessed associations between dietary variables and self-reported diabetes, adjusting for age, sex, body mass index (BMI), education, income, employment, and physical activity. Dietary variables were energy-adjusted using the residual method. **Results**: Higher odds of diabetes were observed among individuals who reported obesity (OR: 2.70; 95% CI: 1.96–3.72), lower educational attainment (OR: 0.73 for high school vs. <high school; 95% CI: 0.55–0.99), and unemployment (OR: 0.66 for employed vs. unemployed; 95% CI: 0.46–0.95). Lower odds were observed among participants reporting regular physical activity (OR: 0.59; 95% CI: 0.43–0.81) and less frequent processed meat consumption (OR: 0.53; 95% CI: 0.37–0.76). Inverse associations were also observed for moderate dessert consumption, whereas strong positive associations were identified for adherence to special diets (OR: 9.24; 95% CI: 7.09–12.06) and dietitian consultation (OR: 6.30; 95% CI: 4.13–9.61). **Conclusions**: In this nationally representative cross-sectional analysis, dietary patterns and sociodemographic factors were statistically associated with diabetes prevalence. The results should be interpreted cautiously, as observed associations may reflect behavioral changes following diagnosis rather than causal relationships. Prospective studies are required to clarify temporal direction and underlying mechanisms.

## 1. Introduction

Annually, 90% of deaths in Europe are attributed to chronic, non-communicable diseases [1]. Premature mortality caused by cardiovascular diseases, cancer, diabetes mellitus, and chronic respiratory diseases accounts for 68% of all premature deaths in Europe. These conditions represent approximately 80% of the healthcare burden on countries, and their prevalence continues to increase globally [2].

The incidence of non-communicable chronic diseases is uneven across populations, with those with low socioeconomic status being more severely affected. In addition, complications and the need for insulin therapy are more common among diabetic patients with lower educational levels, which suggests that diagnosis and early treatment of the disease are not effective enough [3,4,5].

Diabetes is currently one of the most common non-communicable diseases in Europe, affecting 7% of the population, translating to more than 33 million people, while globally, it affects 537 million individuals. The prevalence of diabetes among the Hungarian population aged 20–69 is 7.47% [95% CI, 6.26–8.69], while the prevalence of IFG [impaired fasting glucose, fasting blood glucose: 6.1–6.9 mmol/L] in this group is 4.39% [95% CI, 3.44–5.34] [6]. Chronic non-communicable diseases, such as diabetes mellitus, overweight, obesity, and endocrine disorders, are increasingly affecting children as well; the adjusted incidence of diabetes among children and adolescents nearly doubled in recent decades [7].

Besides this, many people live with undiagnosed diabetes mellitus. According to a study, the prevalence of undiagnosed diabetes can be approximately 3% of the whole population [8]. A nationally representative study showed a similar rate in Hungary, with 2.2% of women and 2.8% of men having undiagnosed diabetes [9]. Consequently, behavioral risk factors play a significant role in the number of lost healthy life years and the financial burden on countries [10].

Diabetes is a group of physiological dysfunctions characterized by hyperglycemia resulting from insulin resistance, inadequate insulin secretion, or excessive glucagon secretion. Type 1 diabetes is an autoimmune disease that leads to the destruction of pancreatic beta cells [11]. Type 2 diabetes, which is much more common, is primarily caused by a combination of pancreatic beta-cell dysfunction and insulin resistance, leading to progressively impaired glucose regulation [12]. Research findings indicate that diabetes can damage entire organ systems, particularly blood vessels, the eyes, the kidneys, the heart, and nerves. If left untreated or improperly managed, diabetes can lead to complications such as diabetic nephropathy, diabetic neuropathy, and diabetic retinopathy [13].

Poor glycemic control is most strongly associated with unhealthy dietary habits. The increasing consumption of processed and high-sugar foods, including fast food and sugary beverages, significantly raises the risk of diabetes, as do irregular eating patterns and skipped breakfasts [14]. According to most studies, a plant-based diet, increased consumption of vegetables and fruits, and higher education levels serve as protective factors [15,16]. These correlations have been established through the analysis of HbA1c levels across different groups. Other research has demonstrated that unemployment and low income are also linked to unhealthy eating habits and food insecurity [17].

Dietary determinants are assumed to consistently play a central role in the presentation of diabetes. Diabetes is a prevalent disease worldwide typically brought about by an energy-dense high-fat diet, combined with a sedentary lifestyle [18]. As current macronutrient intake is shown to be characterized by a high ratio of fat and monounsaturated fats, results have suggested that diets high in monounsaturated fats, poultry, and full-fat dairy products are typically known to be determinative in the development of diabetes. Furthermore, diet is assumed to consistently play a key role in the presentation of diabetes. The high prevalence of type 2 diabetes has emerged in the context of the transition of dietary patterns along with changes in lifestyle factors, socioeconomic conditions and urbanization, particularly with respect to less developed countries. Whereas industrialized countries are known to resort to consuming refined sugars and high-fat, processed food together with having a higher caloric intake, the typical diet of underdeveloped countries is characterized primarily by high fiber, low caloric intake, and lean animal products. With the ongoing transition of dietary patterns, these countries may also increasingly tend to strengthen energy-dense and high-fat diets, ultimately contributing to increasing levels of obesity, unhealthy eating habits and less physical activity, which are the main sources of diabetes. Since the mid-1980s, the prevalence of diabetes in Hungary has increased more rapidly than the global average annual growth rate and has shown marked upward trends compared to several Central and Eastern European countries. Moreover, projections from the World Health Organization have suggested a substantial continued increase in diabetes prevalence in Hungary between 2000 and 2030 [19,20,21,22,23,24].

This study aimed to use data from the Hungarian European Health Interview Survey (EHIS) 2019 to examine the associations between the frequency of food consumption and the presence of diabetes in the Hungarian population, and to explore how sociodemographic characteristics and lifestyle-related factors are related to these associations.

Although several studies have examined diabetes prevalence in Hungary, population-level evidence specifically addressing habitual dietary patterns in relation to diabetes using nationally representative data remains limited. This gap warrants investigation given potential country-specific dietary habits and policy contexts.

To our knowledge, this is the first study in Hungary and the broader Central and Eastern European region to use nationally representative EHIS data to systematically examine the association between habitual dietary frequency variables and diabetes prevalence within a unified multivariable framework. By integrating dietary, socioeconomic, and behavioral factors in a single weighted analysis, the present study provides contemporary population-level evidence from a harmonized European data source.

## 2. Materials and Methods

### 2.1. Study Population and Data Source

This cross-sectional study utilized data from the 2019 wave of the Hungarian EHIS, which is a nationally representative, population-based survey conducted across the Member States of the European Union, adhering to a harmonized methodology that collects information on health status, health behaviors, and sociodemographic characteristics of individuals aged 15 years and older residing in private households. The sampling was based on a stratified, multistage probability design. Sampling weights were calculated following the methodological guidelines of Eurostat, taking into account the probability of selection, adjustments for non-response, and calibration to align with the national population distribution by sex, age, region, and degree of urbanization [25,26,27,28,29].

The final sample comprised 5603 participants aged 15 years or older at the time of data collection. Data were gathered using a standardized Eurostat questionnaire administered through a combination of face-to-face and computer-assisted personal interviews. The Hungarian Central Statistical Office was responsible for data collection and processing, while the dataset was made accessible to the research team by the Department of Epidemiology at the University of Debrecen. All data were fully anonymized, and the study adhered to the ethical principles outlined in the Declaration of Helsinki.

### 2.2. Variable Definitions and Measurement

The primary outcome variable was self-reported diabetes mellitus, recorded in a binary form (yes/no). The explanatory variables encompassed sociodemographic, lifestyle, and dietary factors. Sociodemographic variables included age, sex, type of residence, educational attainment, employment status, subjective financial status, and objective income quintiles. Data were derived from self-reported height and weight, which were used to calculate body mass index (BMI, kg/m^2^) and classify individuals as normal weight, overweight, or obese. Lifestyle variables comprised smoking status, alcohol consumption, engagement in at least 10 min of moderate or vigorous physical activity per week, daily intake of vegetables and desserts, and frequency of consumption of red meat, white meat, processed meat, and fish. Alcohol consumption was derived from the original EHIS frequency categories and recoded into a binary variable. Participants reporting less than one alcoholic drink per month were classified as non-drinkers, whereas those reporting one or more drinks per month (including 1–3 drinks per month, 1–4 drinks per week, or daily/almost daily consumption) were categorized as drinkers.

Physical activity was defined according to the EHIS item assessing engagement in at least 10 min of moderate or vigorous physical activity per week and was analyzed as a binary variable (yes/no).

Energy drink consumption was also recoded into a binary variable. Participants reporting consumption more than once per week were classified as regular consumers, while those reporting consumption once per week or less, including never, were categorized as non-consumers.

Dietary intake variables were assessed using self-reported frequency categories provided in the EHIS questionnaire. Daily vegetable consumption was categorized as less than one portion per day, one portion per day, two portions per day, or three portions per day. Dessert consumption was categorized as at least one portion per day, less than one portion per day, or rarely/no consumption.

Red meat consumption was classified into four categories: daily or almost daily, 2–3 times per week, once per week, and less than once per week or no consumption. White meat consumption was categorized as daily or almost daily, 2–3 times per week, or once per week or less. Processed meat consumption was categorized as every day, 4–6 times per week, 2–3 times per week, or once per week or less. Fish consumption was categorized as never, less than once per week, or regularly once per week.

Daily water intake was categorized as less than 1 L per day, 1–1.5 L per day, 1.5–2 L per day, or more than 2 L per day.

Additional lifestyle factors included the practice of adding salt to food after cooking, adherence to a special diet and consultations with a dietitian. The coding and categorization of variables were performed in line with the methodology applied in previous publications of the faculty’s researchers [30,31,32,33].

### 2.3. Statistical Analysis

All analyses employed survey weights to account for the complex sampling design, ensuring national representativeness. Descriptive statistics were reported as weighted frequencies and percentages for categorical variables. Associations between groups were assessed using the weighted Pearson’s chi-square test. For weighted logistic regression models, odds ratios (ORs) and 95% confidence intervals (CIs) were calculated, adjusting for potential confounders, while model robustness was confirmed. Model fit was evaluated using the Hosmer–Lemeshow goodness-of-fit test, with model selection determined by the Akaike Information Criterion (AIC) and Bayesian Information Criterion (BIC). Statistical significance was set at a two-sided *p*-value of less than 0.05. All analyses were conducted using STATA IC version 18.0 [34]. Figures were generated using Python (version 3.10.12) with the Matplotlib library (version 3.7.1).

## 3. Results

The EHIS collected data from 5603 participants. Table 1 presents descriptive statistics for individuals with and without diabetes. Individuals aged 65 and over had a higher prevalence of diabetes (50.97%, *p* < 0.001) compared to younger age groups.

Educational attainment was associated with diabetes prevalence: participants with education levels below high school exhibited higher diabetes rates (52.94%, *p* < 0.001). Unemployment was significantly associated with an increased prevalence of diabetes (67.71%, *p* < 0.001). Financial status and income quintiles also showed relevance to diabetes prevalence: participants with an average financial status had a higher proportion of diabetes (60.41%, *p* = 0.0176), while those in the low-income quintile reported higher diabetes prevalence (*p* = 0.009). Body mass index (BMI) was a strong predictor of diabetes, with obese individuals reporting significantly higher rates of the condition (45.75%, *p* < 0.001). Lifestyle factors, including smoking status, alcohol consumption, self-perceived health, use of over-the-counter (OTC) medications or supplements, exercise habits, and dietary patterns, also showed significant correlations with diabetes prevalence. Smokers and alcohol consumers reported lower diabetes rates (*p* = 0.008 and *p* < 0.001, respectively). The absence of at least 10 min of weekly exercise was associated with a higher prevalence of diabetes (*p* < 0.001). Daily vegetable intake and dessert consumption patterns were linked to diabetes prevalence (*p* = 0.0246 and *p* < 0.001, respectively). Water consumption was also associated with diabetes risk: individuals consuming less than 1 L per day exhibited a lower prevalence of the condition (*p* = 0.002). Following a specific diet and consulting a dietitian were significantly related to higher diabetes prevalence (*p* < 0.001 for both).

Energy drink consumption was also significantly associated with diabetes prevalence (*p* < 0.001), with diabetic individuals reporting a higher proportion of energy drink consumption compared to nondiabetic individuals (Table 1).

**Figure 1:**
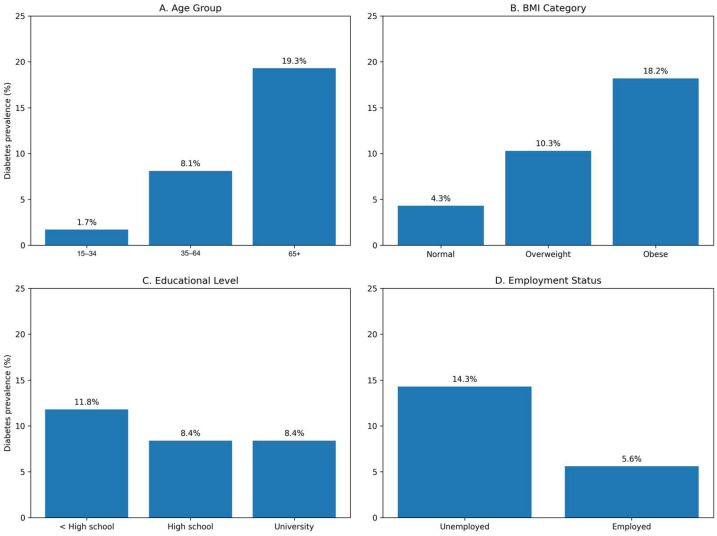
Crude prevalence (%) of self-reported diabetes across selected risk factor categories in the Hungarian European Health Interview Survey (EHIS) 2019 sample. Panels show prevalence by (**A**) age group, (**B**) body mass index (BMI) category, (**C**) educational level, and (**D**) employment status. Prevalence was calculated as the proportion of individuals with self-reported diabetes within each category.

The crude prevalence of self-reported diabetes across key sociodemographic and clinical categories is illustrated in Figure 1. Higher prevalence was observed among older age groups and individuals with obesity, whereas lower prevalence was seen among younger participants, those with normal BMI, and employed individuals.

In the logistic regression analysis indicated in Table 2, participants aged 15–34 (OR: 0.24, 95% CI: 0.13–0.45) and 35–64 (OR: 0.61, 95% CI: 0.42–0.87) had lower odds of diabetes compared to those aged 65 and above. Having a high school education (OR: 0.73, 95% CI: 0.55–0.99) was associated with reduced odds of diabetes compared to those with lower than high school education. Employed individuals had lower odds of diabetes (OR: 0.66, 95% CI: 0.46–0.95) compared to unemployed participants. Urban residence was associated with increased odds of diabetes (OR: 1.31, 95% CI: 1–1.71). Higher-BMI categories, overweight (OR: 1.58, 95% CI: 1.15–2.18) and obese (OR: 2.7, 95% CI: 1.96–3.72), were significantly associated with increased odds of diabetes. Consuming less than one portion of dessert per day (OR: 2.01, 95% CI: 1.34–3) and rarely or not consuming desserts (OR: 2.21, 95% CI: 1.62–3.01) were associated with higher odds of diabetes. Taking OTC drugs or supplements was associated with reduced odds of diabetes (OR: 0.48, 95% CI: 0.37–0.63). Participants who exercised at least 10 min per week had lower odds of diabetes (OR: 0.59, 95% CI: 0.43–0.81). Consuming processed meat once a week or even less was associated with lower odds of diabetes (OR: 0.53, 95% CI: 0.37–0.76). Following a specific diet (OR: 9.24, 95% CI: 7.09–12.06) and regularly consulting a dietitian (OR: 6.3, 95% CI: 4.13–9.61) were associated with higher odds of diabetes. Regular consumption of energy drinks was associated with increased odds of diabetes (OR: 1.63, 95% CI: 1.03–2.59) (Table 2).

## 4. Discussion

The prevalence of diabetes is associated with demographic, socioeconomic, and lifestyle characteristics. In this nationally representative cross-sectional analysis, several dietary behaviors were statistically associated with diabetes prevalence, including lower processed meat intake, regular physical activity, and moderate dessert consumption, while adherence to special diets and dietitian consultations showed positive associations. These findings are consistent with the previous literature describing associations between these factors and diabetes prevalence and may help identify subgroups requiring targeted public health attention.

Given the cross-sectional design, all observed associations should be interpreted cautiously, as reverse causation and post-diagnosis behavioral modification cannot be excluded. In particular, individuals diagnosed with diabetes are likely to modify their dietary behaviors following medical advice or self-management efforts. Such post-diagnosis changes may substantially influence reported consumption patterns, potentially generating inverse or exaggerated associations in cross-sectional analyses. Therefore, the directionality of several dietary findings cannot be determined within the present design. Cross-sectional nutritional epidemiology is particularly vulnerable to reverse causation and reporting bias, especially for conditions such as diabetes that require long-term dietary management. Individuals aware of their diagnosis may consciously modify their intake of specific food groups, selectively reduce foods perceived as unhealthy, or differentially report consumption in line with medical advice. As a result, observed associations may reflect behavioral adaptation rather than etiological relationships. This methodological limitation should be carefully considered when interpreting counterintuitive findings [35].

Age showed a strong association with diabetes prevalence, with lower odds observed among younger age groups. This finding aligns with prior research [36,37] and reflects well-established age-related metabolic and endocrine changes that increase susceptibility in older populations [38,39].

Educational attainment was associated with diabetes prevalence, with lower odds observed among participants with at least high school education. This pattern is consistent with previous research linking lower education to differences in health literacy, access to healthcare, and lifestyle behaviors [40,41].

The findings indicate that being employed is associated with reduced odds of diabetes relative to being unemployed. Previous research has reported associations between unemployment, psychological stress, and higher diabetes prevalence [42,43]. Furthermore, financial constraints associated with unemployment have been linked to differences in access to healthier food options and healthcare services.

Income quintiles were not significantly associated with diabetes prevalence in our model. However, global evidence suggests [44,45] socioeconomic gradients in diabetes prevalence across multiple regions [46].

Urban residence was associated with higher diabetes prevalence, although the magnitude was modest. Similar patterns have been reported in prior studies, often discussed in relation to differences in lifestyle environments and healthcare access [47].

Our findings are consistent with the well-established association between obesity and diabetes. This association is biologically plausible [48,49], as excess adipose tissue promotes insulin resistance and metabolic dysregulation through inflammatory and hormonal pathways [50,51].

Interestingly, energy drink consumption was positively associated with diabetes prevalence. This finding is consistent with previous studies reporting associations between sugar-sweetened beverage intake and diabetes prevalence [52,53]. Excessive sugar consumption has been linked to insulin resistance, inflammation, and pancreatic beta-cell dysfunction in experimental and longitudinal research. Beyond sugar content, energy drinks may be part of broader behavioral patterns associated with metabolic risk [54,55], including irregular dietary habits and sedentary behavior [56,57]. These findings may reflect a broader cluster of lifestyle characteristics associated with diabetes rather than a direct effect of energy drink consumption itself. Consequently, this association should not be interpreted as evidence of a causal effect of energy drink consumption on diabetes prevalence within this cross-sectional framework.

In Hungary, a broad Public Health Product Tax targeting foods and beverages with high sugar content, including sugar-sweetened beverages, soft drinks, and energy drinks, was introduced in September 2011 with the aim of discouraging consumption of unhealthy products and improving population nutrition. Although some short-term reductions in purchases of taxed products were observed following implementation, longer-term evidence suggests limited sustained decreases in overall sweetened beverage consumption at the population level. Taken together with our finding that regular consumption of energy drinks was statistically associated with diabetes prevalence, these contextual insights underscore the complexity of modifying beverage consumption patterns through fiscal policy alone. They also emphasize that cross-sectional associations in our study likely reflect broader lifestyle and dietary patterns rather than direct effects of single policies on diabetes outcomes [58].

Conversely, regular physical activity was associated with lower odds of diabetes in this cross-sectional analysis. Even minimal physical activity was associated with lower diabetes prevalence, consistent with established evidence linking physical activity to improved metabolic regulation [59,60].

Associations between certain dietary patterns and diabetes prevalence were observed in our study. Moderate dessert consumption was associated with a lower prevalence of diabetes. While this finding contrasts with conventional studies linking sugar intake to diabetes risk [61], it may reflect broader dietary patterns or behavioral characteristics not fully captured in the model. Alternatively, this association may reflect reverse causality, whereby individuals who have already been diagnosed with diabetes or prediabetes reduce their dessert intake following medical advice or self-regulation. Several studies have highlighted that dietary reporting in cross-sectional surveys may be influenced by disease status, with individuals underreporting or altering their intake of ‘unhealthy’ foods after diagnosis [62,63]. In particular, diagnosed patients often adopt restrictive dietary behaviors, such as reduced consumption of sweets and high-glycemic foods, which can lead to an apparent inverse association between these items and disease prevalence [64]. This underscores the need for longitudinal studies to more accurately determine the directionality of such associations. Importantly, this inverse association should not be interpreted as evidence that dessert consumption confers any protective or beneficial effect. Rather, it is highly plausible that individuals with diagnosed diabetes reduce their intake of sweets, resulting in an apparent statistical association driven by dietary restriction following diagnosis.

Similarly, higher processed meat consumption was statistically associated with higher diabetes prevalence, whereas lower intake was associated with lower odds. This finding is consistent with studies suggesting that processed meats contribute to insulin resistance and metabolic dysfunction [65,66]. Processed meat is typically defined as meat that has been preserved by smoking, curing, salting, or the addition of chemical preservatives, and includes products such as sausages, hot dogs, bacon, ham, and salami. These products often contain high levels of saturated fat, sodium, nitrates, nitrites, and advanced glycation end products (AGEs), all of which have been implicated in promoting oxidative stress, systemic inflammation, and impaired insulin signaling [67,68]. Furthermore, several meta-analyses have shown a robust association between processed meat intake and increased risk of type 2 diabetes, even after adjusting for body mass index and total energy intake [69,70]. These findings are in line with existing evidence and dietary recommendations that advise limiting processed meat consumption.

Finally, following a special diet and consulting a dietitian were associated with a higher prevalence of diabetes in our study. This most plausibly reflects reverse causation, as individuals diagnosed with diabetes are frequently advised to adopt specific dietary regimens and seek professional nutritional counseling. Therefore, these associations likely represent post-diagnosis management behaviors rather than factors contributing to diabetes development.

Overall, given the cross-sectional nature of the data and the reliance on self-reported dietary behaviors, the observed dietary associations should be interpreted as hypothesis-generating rather than causal.

A major strength of this study is the use of nationally representative data from the Hungarian EHIS 2019, which supports the generalizability of the findings to the population of Hungary. The large sample size and the availability of detailed sociodemographic, lifestyle, and dietary information enabled multivariable analyses of outcomes. The standardized EHIS data collection methodology facilitates comparison with findings from other countries using the same survey framework.

Several limitations should also be considered. Due to the cross-sectional study design, the results should be interpreted as associations, and causal relationships cannot be inferred. Reverse causation cannot be excluded. All variables were self-reported, including diabetes diagnosis and dietary exposures, which may introduce recall, reporting or misclassification bias. Dietary intake was assessed based on consumption frequency without information on portion size, potentially leading to exposure misclassification. Moreover, dietary data were based on self-reported frequency categories rather than quantitative intake measures, limiting the ability to assess dose–response relationships. The absence of detailed portion size information and potential differential reporting following diagnosis may have introduced measurement error and attenuated or distorted observed associations.

Residual confounding from unmeasured factors remains possible, and some health outcomes lacked clinical validation, which may affect measurement precision. Future longitudinal or interventional studies are needed to further explore the observed associations.

## 5. Conclusions

This study demonstrates that obesity, low educational attainment, unemployment, and unhealthy dietary patterns are strongly associated with diabetes mellitus prevalence in Hungary. Regular physical activity and reduced processed meat intake were protective, while adherence to special diets and dietitian consultations likely reflected post-diagnosis adaptations rather than causal risk factors. Public health strategies should prioritize early prevention through targeted nutritional education, obesity prevention, and promotion of active lifestyles, especially among vulnerable groups. Policy measures limiting the consumption of processed meat and energy drinks could further reduce diabetes risk. Future longitudinal and intervention studies are essential to confirm causal relationships and guide effective, evidence-based prevention programs.

## Figures and Tables

**Table 1:** Sociodemographic lifestyle. Dietary and health-related characteristics of participants with and without diabetes.

Characteristics		Diabetes			
		Without Diabetes (*n.* %)	With Diabetes (*n*. %)	Total (*n.* %)	*p*-Values
**Gender**	Male	2279 (47.09)	256 (47.52)	2535 (47.13)	0.8575
	Female	2704 (52.91)	294 (52.48)	2998 (52.87)	
**Age groups**	65+	1303 (20.51)	312 (50.97)	1615 (23.21)	**<0.001**
	15–34	1245 (29.36)	22 (5.31)	1267 (27.23)	
	35–64	2435 (50.13)	216 (43.72)	2651 (49.56)	
**Educational levels**	Lower than high school	2195 (41.32)	294 (52.94)	2489 (42.35)	**<0.001**
	High school	1660 (34.19)	153 (27.95)	1813 (33.64)	
	University	1128 (24.49)	103 (19.11)	1231 (24.02)	
**Employment**	Unemployed	2378 (43.19)	396 (67.71)	2774 (45.36)	**<0.001**
	Employed	2605 (56.81)	154 (32.29)	2759 (54.64)	
**Residence area**	Rural	1646 (30.18)	155 (26.94)	1801 (29.9)	0.125
	Urban	3337 (69.82)	395 (73.06)	3732 (70.1)	
**Financial status**	Average	2775 (55.5)	328 (60.41)	3103 (55.94)	**0.018**
	Good	1516 (33.11)	141 (26.71)	1657 (32.54)	
	Bad	587 (11.39)	68 (12.88)	655 (11.52)	
**Income quintiles**	Low	1034 (20.05)	114 (21.52)	1148 (20.18)	**0.009**
	Lower middle	1033 (19.89)	132 (22.5)	1165 (20.12)	
	Middle	1011 (19.93)	114 (20.98)	1125 (20.02)	
	Higher middle	1121 (22.3)	128 (23.7)	1249 (22.43)	
	High	784 (17.82)	62 (11.3)	846 (17.24)	
**BMI**	Normal	2093 (44.2)	94 (18.48)	2187 (41.93)	**<0.001**
	Overweight	1716 (34.08)	196 (35.77)	1912 (34.23)	
	Obese	1126 (21.72)	251 (45.75)	1377 (23.84)	
**Smoking**	Smoker	1402 (28.46)	115 (22.65)	1517 (27.95)	**0.008**
	Non-smoker	3581 (71.54)	435 (77.35)	4016 (72.05)	
**Alcohol use**	Drinker	3507 (72.01)	342 (62.01)	3849 (71.12)	**<0.001**
	Non-drinker	1476 (27.99)	208 (37.99)	1684 (28.88)	
**Self-perceived health**	Average	1444 (26.3)	283 (50.87)	1727 (28.47)	**<0.001**
	Good	3048 (65.55)	120 (23.35)	3168 (61.81)	
	Bad	474 (8.15)	145 (25.78)	619 (9.71)	
**Taking OTC drugs or supplements**	No	983 (18.12)	220 (40.77)	1203 (20.14)	**<0.001**
	Yes	3979 (81.88)	329 (59.23)	4308 (79.86)	
**Level of daily activity**	Inactive	1883 (41.24)	217 (43.35)	2100 (41.43)	0.379
	Active	2881 (58.76)	308 (56.65)	3189 (58.57)	
**Exercising at least 10 min per week**	No	2895 (55.55)	410 (74.9)	3305 (57.25)	**<0.001**
	Yes	2022 (44.45)	129 (25.1)	2151 (42.75)	
**Daily vegetable consumption**	Less than 1 portion	2738 (55.69)	262 (48.44)	3000 (55.05)	**0.024**
	1 portion	1135 (22.66)	144 (25.66)	1279 (22.92)	
	2 portions	730 (14.83)	95 (17.98)	825 (15.11)	
	3 portions	329 (6.82)	39 (7.91)	368 (6.91)	
**Dessert portions per day**	At least 1 portion per day	1746 (35.43)	94 (17.15)	1840 (33.81)	**<0.001**
	Less than 1 portion per day	854 (17.59)	84 (14.59)	938 (17.32)	
	Rarely or no consumption	2353 (46.98)	367 (68.26)	2720 (48.87)	
**Red meat consumption**	Daily or almost daily	617 (13.01)	67 (12.43)	684 (12.96)	0.819
	2–3 times per week	1842 (36.71)	200 (36.7)	2042 (36.71)	
	1 time per week	1136 (22.98)	122 (21.76)	1258 (22.87)	
	Less than 1 time per week or no consumption	1345 (27.29)	155 (29.11)	1500 (27.46)	
**White meat consumption**	Daily or almost daily	1158 (24.51)	118 (22.34)	1276 (24.32)	0.162
	2–3 times per week	2891 (57.99)	313 (56.81)	3204 (57.88)	
	Once a week or less	912 (17.51)	112 (20.84)	1024 (17.8)	
**Processed meat consumption**	Every day	1416 (29.41)	167 (31.38)	1583 (29.59)	0.189
	4–6 times per week	1137 (23.88)	105 (20.23)	1242 (23.55)	
	2–3 times per week	1438 (28.29)	173 (31.2)	1611 (28.55)	
	Once a week or even less	969 (18.42)	99 (17.19)	1068 (18.31)	
**Fish consumption**	Never	687 (13.66)	70 (13.27)	757 (13.63)	0.703
	Less than once a week	2912 (58.21)	328 (60.16)	3240 (58.38)	
	Regularly once a week	1345 (28.12)	143 (26.58)	1488 (27.99)	
**Daily water consumption**	Less than 1 L per day	464 (9.18)	30 (6.19)	494 (8.92)	**0.002**
	1–1.5 L per day	807 (16.11)	70 (12.48)	877 (15.78)	
	1.5–2 L per day	1405 (27.92)	146 (26.22)	1551 (27.77)	
	<2 L per day	2286 (46.79)	300 (55.11)	2586 (47.53)	
**Adding salt to food after cooking and serving**	Yes	1490 (30.33)	161 (29.18)	1651 (30.23)	0.599
	No	3459 (69.67)	382 (70.82)	3841 (69.77)	
**Following a specific diet (intermittent fasting, ketogenic, et** **c.)**	No	4283 (85.27)	238 (42.36)	4521 (81.46)	**<0.001**
	Yes	700 (14.73)	312 (57.64)	1012 (18.54)	
**Do you regularly consult a dietitian**	No	4864 (97.68)	439 (79.02)	5303 (96.03)	**<0.001**
	Yes	114 (2.32)	110 (20.98)	224 (3.97)	
**Regular energy drink consumption**	No	986 (21.77)	34 (7.27)	1020 (20.48)	**<0.001**
	Yes	3968 (78.23)	510 (92.73)	4478 (79.52)	
**How much can you do for your health**	Very little or nothing	810 (14.86)	154 (26.79)	964 (15.92)	**<0.001**
	I can do a few things	2860 (57.87)	309 (57.59)	3169 (57.84)	
	I can do a lot	1253 (27.27)	80 (15.62)	1333 (26.24)	

Bold values indicate statistical significance (*p* < 0.05) based on weighted Pearson’s chi-squared tests.

**Table 2:** Weighted multiple logistic regression analysis of factors affecting diabetes prevalence.

Characteristics		Diabetes	
		OR (95% CI)	*p*-Value
**Gender**	Male	reference	
	Female	0.8 [0.61–1.04]	0.097
**Age groups**	65+	reference	
	15–34	**0.24 [0.13–0.45]**	**<0.001**
	35–64	**0.61 [0.42–0.87]**	**0.007**
**Educational levels**	Lower than high school	reference	
	High school	**0.73 [0.55–0.99]**	**0.039**
	University	0.68 [0.46–1.01]	0.053
**Employment**	Unemployed	reference	
	Employed	**0.66 [0.46–0.95]**	**0.026**
**Residence area**	Rural	reference	
	Urban	**1.31 [1–1.71]**	**0.048**
**Financial status**	Average	reference	
	Good	1.14 [0.85–1.54]	0.379
	Bad	0.75 [0.52–1.09]	0.128
**Income quintiles**	Low	reference	
	Lower middle	0.94 [0.66–1.33]	0.72
	Middle	1.05 [0.72–1.54]	0.785
	Higher middle	1.36 [0.93–2]	0.117
	High	1.35 [0.8–2.25]	0.259
**BMI**	Normal	reference	
	Overweight	**1.58 [1.15–2.18]**	**0.005**
	Obese	**2.7 [1.96–3.72]**	**<0.001**
**Smoking**	Smoker	reference	
	Non-smoker	0.86 [0.64–1.15]	0.31
**Alcohol use**	Drinker	reference	
	Non-drinker	1.01 [0.76–1.33]	0.964
**Self-perceived health**	Average	reference	
	Good	**0.47 [0.35–0.65]**	**<0.001**
	Bad	1.32 [0.94–1.86]	0.114
**Taking OTC drugs or supplements**	No	reference	
	Yes	**0.48 [0.37–0.63]**	**<0.001**
**Level of daily activity**	Inactive	reference	
	Active	0.96 [0.75–1.24]	0.762
**Exercising at least 10 min per week**	No	reference	
	Yes	**0.59 [0.43–0.81]**	**0.001**
**Daily vegetable consumption**	Less than 1 portion	reference	
	1 portion	0.92 [0.69–1.23]	0.566
	2 portions	1.02 [0.71–1.45]	0.924
	3 portions	0.9 [0.55–1.46]	0.659
**Dessert portions per day**	At least 1 portion per day	reference	
	Less than 1 portion per day	**2.01 [1.34–3]**	**0.001**
	Rarely or no consumption	**2.21 [1.62–3.01]**	**<0.001**
**Red meat consumption**	Daily or almost daily	reference	
	2–3 times per week	0.92 [0.61–1.39]	0.7
	1 time per week	0.87 [0.56–1.36]	0.542
	Less than 1 time per week or no consumption	1 [0.65–1.56]	0.993
**White meat consumption**	Daily or almost daily	reference	
	2–3 times per week	1.04 [0.76–1.42]	0.823
	Once a week or less	1.05 [0.72–1.54]	0.803
**Processed meat consumption**	Every day	reference	
	4–6 times per week	0.76 [0.53–1.08]	0.129
	2–3 times per week	0.86 [0.63–1.17]	0.347
	Once a week or even less	**0.53 [0.37–0.76]**	**0.001**
**Fish consumption**	Never	reference	
	Less than once a week	1.13 [0.78–1.64]	0.527
	Regularly once a week	1 [0.65–1.54]	0.995
**Daily water consumption**	Less than 1 L per day	reference	
	1–1.5 L per day	0.88 [0.48–1.59]	0.666
	1.5–2 L per day	0.98 [0.58–1.66]	0.932
	<2 L per day	1.36 [0.81–2.26]	0.243
**Adding salt to food after cooking and serving**	Yes	reference	
	No	0.95 [0.72–1.26]	0.717
**Following a specific diet (intermittent fasting, ketogenic, etc** **.)**	No	reference	
	Yes	**9.24 [7.09–12.06]**	**<0.001**
**Do you regularly consult a dietitian**	No	reference	
	Yes	**6.3 [4.13–9.61]**	**<0.001**
**How much can you do for your health**	Very little or nothing	reference	
	I can do a few things	0.97 [0.71–1.32]	0.823
	I can do a lot	**0.6 [0.39–0.93]**	**0.022**
**Regular energy drink consumption**	Yes	reference	
	No	**1.63 [1.03–2.59]**	**0.038**

Bold values indicate statistical significance (*p* < 0.05). The ORs (odds ratios) and 95% CIs (confidence intervals) are adjusted for other variables in the model.

## Data Availability

The data analyzed in this study are subject to the following licenses/restrictions: The data presented in this study are available upon request from Hungarian Central Statistical Office, which performed and supervised the data collection. Requests to access these datasets should be directed to the Hungarian Central Statistical Office, www.ksh.hu/?lang=en (accessed on 14 July 2025).
